# An Analysis of 332 Fatalities Infected with Pandemic 2009 Influenza A (H1N1) in Argentina

**DOI:** 10.1371/journal.pone.0033670

**Published:** 2012-04-10

**Authors:** Ana M. Balanzat, Christian Hertlein, Carlos Apezteguia, Pablo Bonvehi, Luis Cámera, Angela Gentile, Oscar Rizzo, Manuel Gómez-Carrillo, Fatima Coronado, Eduardo Azziz-Baumgartner, Pollyanna R. Chávez, Marc-Alain Widdowson

**Affiliations:** 1 National Ministry of Health of Argentina, Buenos Aires, Argentina; 2 Sociedad Argentina de Terapia Intensiva, Buenos Aires, Argentina; 3 Sociedad Argentina de Infectología, Capital Federal, Argentina; 4 Sociedad Argentina de Medicina, Buenos Aires, Argentina; 5 Sociedad Argentina de Pediatría, Buenos Aires, Argentina; 6 Asociación Argentina de Medicina Respiratoria, Buenos Aires, Argentina; 7 Asociación Argentina de Microbiología, Buenos Aires, Argentina; 8 Influenza Division, Centers for Disease Control and Prevention, Atlanta, Georgia, United States of America; University of Giessen Lung Center, Germany

## Abstract

**Background:**

The apparent high number of deaths in Argentina during the 2009 pandemic led to concern that the influenza A H1N1pdm disease was different there. We report the characteristics and risk factors for influenza A H1N1pdm fatalities.

**Methods:**

We identified laboratory-confirmed influenza A H1N1pdm fatalities occurring during June-July 2009. Physicians abstracted data on age, sex, time of onset of illness, medical history, clinical presentation at admission, laboratory, treatment, and outcomes using standardize questionnaires. We explored the characteristics of fatalities according to their age and risk group.

**Results:**

Of 332 influenza A H1N1pdm fatalities, 226 (68%) were among persons aged <50 years. Acute respiratory failure was the leading cause of death. Of all cases, 249 (75%) had at least one comorbidity as defined by Advisory Committee on Immunization Practices. Obesity was reported in 32% with data and chronic pulmonary disease in 28%. Among the 40 deaths in children aged <5 years, chronic pulmonary disease (42%) and neonatal pathologies (35%) were the most common co-morbidities. Twenty (6%) fatalities were among pregnant or postpartum women of which only 47% had diagnosed co-morbidities. Only 13% of patients received antiviral treatment within 48 hours of symptom onset. None of children aged <5 years or the pregnant women received antivirals within 48 h of symptom onset. As the pandemic progressed, the time from symptom-onset to medical care and to antiviral treatment decreased significantly among case-patients who subsequently died (p<0.001).

**Conclusion:**

Persons with co-morbidities, pregnant and who received antivirals late were over-represented among influenza A H1N1pdm deaths in Argentina, though timeliness of antiviral treatment improved during the pandemic.

## Introduction

In April 2009, pandemic influenza H1N1 2009 (influenza A H1N1pdm virus emerged in Mexico and the United States [1]. Argentina was one of the first countries to experience pandemic H1N1 virus transmission during the usual influenza season [2], [3]. The first influenza A H1N1pdm-confirmed case was identified on April 28^th^ 2009. This and subsequent cases were among travelers arriving from North America. The virus did not spread in the community until mid-May. The first group affected was school-aged children in Buenos Aires.

On June 15^th^ 2009, the first fatal influenza A H1N1pdm case in Argentina was confirmed [4]. Ten days later, 22 additional influenza A H1N1pdm fatalities were confirmed in the province of Buenos Aires. During July (epidemiological weeks 26–30), the number of laboratory-confirmed influenza A H1N1pdm cases rose from 2,409 to 5,712 and the number of confirmed deaths from 55 to 337 which represented an increase in case-fatality proportion among reported cases from 2.2% to 5.9%. This was the largest number of influenza A H1N1pdm deaths reported from any country at that time. Although the initial elevated mortality was considered a surveillance artifact, concerns remained that influenza A H1N1pdm disease evolution was somehow different in Argentina. Public health officials were also concerned that prevention and treatment measures might not have been sufficiently targeted, given the limited size of local oseltamivir stockpiles for presumptive treatment of case-patients.

On June 26^th^ 2009, the National Ministry of Health of Argentina (NMHA) created the National Commission for the Assessment of Influenza A (H1N1) to assess clinical aspects of influenza A H1N1pdm illness among fatalities, adequacy and timeliness of treatment, and possible risk factors for severe illness [5]. In this report, we describe the features of 332 laboratory-confirmed influenza A H1N1pdm fatalities with available clinical data that occurred during June 15–July 31 2009, at the peak of influenza A H1N1pdm transmission in Argentina.

## Methods

In April 2009, the Government of Argentina activated the national Emergency Situation Room (ESR). Each province reported laboratory and epidemiological data on confirmed influenza A H1N1pdm fatalities and hospitalizations through the National Health Surveillance System and from laboratories performing real-time reverse-transcription polymerase-chain-reaction (rRT-PCR) for influenza A H1N1pdm through the Laboratory Surveillance System [6], [7]. Public and private health institutions were mandated to notify the ESR of hospitalized influenza A H1N1pdm case-patients who subsequently died.

For this study, a confirmed fatal case was defined as a patient who tested positive for influenza A H1N1pdm and who died in the period June 15-July 31 2009 (i.e. the peak of the pandemic). To identify cases, we searched a list of influenza A H1N1pdm laboratory-confirmed fatalities reported to the ESR by private and public institutions from the city of Buenos Aires and the seven provinces with the highest number of reported confirmed cases (i.e. Buenos Aires, Santa Fe, Córdoba, Neuquén, Río Negro, Tucumán, and Entre Ríos).

Physicians abstracted data on age, sex, medical history, comorbidities (i.e. chronic pulmonary, cardiovascular, renal, hepatic, hematological, metabolic, neurologic, immunologic and neonatal disorders), selected risk factors (e.g. obesity, defined as BMI>30 or subjectively assessed; pregnancy, alcoholism, and smoking), previous hospitalizations, signs and symptoms, clinical presentation, diagnosis at admission, duration of hospitalization and intensive care, radiographic findings, use of oseltamivir, and laboratory results using a standardized form. Nosocomial influenza A H1N1pdm virus infection was defined as a patient who developed respiratory symptoms >48 hours after admission for a non-respiratory cause and who later tested positive for influenza A H1N1pdm. Organ failures were defined according to international definitions [8], [9]. If a patient was mechanically ventilated, but not admitted to intensive care unit (ICU) because of space limitations, the case was still classified as an ICU patient. All collected data were reviewed by physicians from the ESR and the National Commission for the Assessment of Influenza A (H1N1) to detect possible inconsistencies. Data was not captured if deaths occurred outside the hospital, the medical records were unavailable, or occurred outside the study period.

Respiratory specimens were collected from suspected cases and were tested for influenza A H1N1pdm by rRT-PCR assay initially in Argentina's National Reference Laboratory. As the pandemic progressed, an additional 18 laboratories were trained to use this technique and provided data for the study.

Differences in categorical variables among the three age groups (i.e.<5, 5–49, and >50 years) were analyzed by Χ^2^ tests and Fisher's exact test. Student t-tests and one-way analysis of variance (ANOVA) were used to compare means and Wilcoxon Rank sum test to compare medians.

### Ethics statement

To maintain data confidentiality, unique identifiers on medical records were coded. Institutional review board approval was not required as the NMHA empowered the commission to confidentially review medical records of influenza A H1N1pdm fatalities through a ministerial resolution [5] during this public health emergency.

## Results

**Figure 1:**
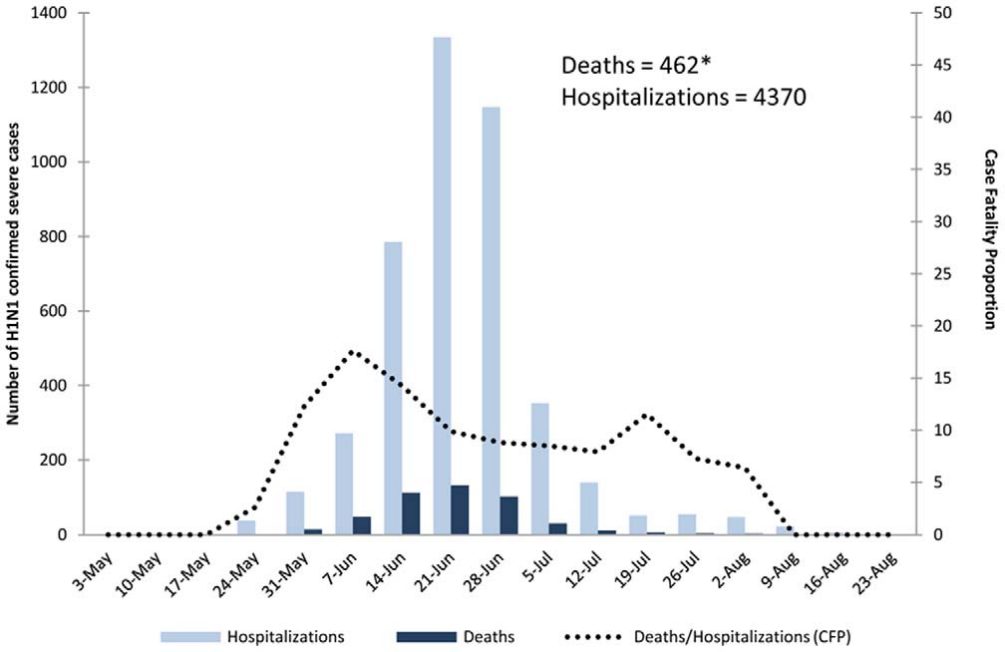
Pandemic H1N1 (influenza A H1N1pdm) confirmed hospitalizations and deaths by week of symptom onset and case fatality proportion among hospitalized cases, Argentina, May 3^rd^ 2009–August 29^th^ 2009.

During 2009, 626 patients died with laboratory-confirmed influenza A H1N1pdm infection in Argentina [10]. The proportion of confirmed hospitalized case-patients who died peaked during June (i.e. 48 [18%] of 272) (Figure 1). Of the 626 fatalities, 377 were reported during June 15-July 31 (the study period). Forty-five were excluded because 37 were not confirmed by RT-PCR and eight had incomplete medical charts.

The remaining 332 fatalities had a median age of 36 years (IQR = 13–53 years) and 177 (53%) were male. Children aged <18 years accounted for 93 (28%) of fatalities, those aged <5 years comprised 48 (52%), and infants aged less <6 months comprised 16 (33%). Although more fatalities were reported among patients aged 19–49 years, laboratory-confirmed influenza A H1N1pdm fatalities per 100,000 population were more common among children aged <5 years and persons aged 50–64 years (Table 1).

**Table 1:** Age and sex distribution of fatalities reported from June 15^th^ to July 31^st^ 2009, Argentina.

	Fatalities reportedN = 332	Fatalities reported (%)	Population* (N)	Population (%)	No. fatalities reported per 100,000 pop
**Age**					
**0 to 4**	48	14	3,349,278	9	1.4
**5 to 18**	45	14	9,442,608	26	0.5
**19 to 49**	134	40	15,241,760	42	0.9
**50 to 64**	75	23	4,638,864	13	1.6
**65+**	30	9	3,587,620	10	0.8
**Sex**					
**Male**	177	53	17,659,072	49	1.0
**Female**	155	47	18,601,058	51	0.8

* Argentina census data (INDEC. Censo Nacional de Población, Hogares y Viviendas 2001).

Thirty fatalities were not hospitalized for influenza symptoms but rather acquired nosocomial influenza A H1N1pdm infections. Excluding these cases, the most common symptoms at admission were dyspnea (94%), cough (93%), and history of fever (85%). During admission, most patients had elevated respiration rates (73%) and heart rates (69%). On auscultation, crackles were more common among fatalities aged ≥5 years compared to fatalities <5 years (89% vs. 63%, p<0.01). Anemia was present in 54% of patients, and more common among those aged <5 years (67%, p<0.01) (Table 2). Of 134 fatalities with available data, 110 (82%) presented with lymphopenia. Of 159 fatalities with available data, 65 (41%) had thrombocytopenia. Elevated urea was more common among patients aged ≥50 compared to the other age groups (p<0.001). Eighty-two percent of patients had oxygen saturation <96%) which was more common among patients ≥5 years (p<0.01); while acidosis (pH<7.36) and elevated pCO2 (>44 mmHg) were more common among patients <5 years (p<0.01) (Table 3).

**Table 2:** Selected symptoms and physical signs at time of admission by age group in influenza A H1N1pdm confirmed fatalities (excluding patients with influenza A H1N1pdm nosocomial infections unless otherwise reported), Argentina, June 15^th^–July 31^st^, 2009.

Age groups (years)	<5N = 48	5–49N = 179	50+N = 105	All agesN = 332
	N/total num of charts with data (%)*			
**influenza A H1N1pdm nosocomial infections**	7/48 (13)	17/179 (9)	6/105 (6)	30/332 (8)
**Symptoms reported at presentation**				
**Fever**	25/30 (83)	118/132 (89)	59/76 (78)	202/238 (85)
**Dyspnea**	34/38 (89)	133/141 (94)	87/91 (96)	254/270 (94)
**Cough**	14/19 (75)	124/133 (93)	84/88 (96)	222/240 (93)
**Gastrointestinal**	4/21 (19)	34/96 (35)	13/66 (20)	51/183 (28)
**Headache**	1/4 (25)	28/48 (58)	15/39 (38)	44/91 (48)
**Sore throat**	1/6 (17)	18/55 (33)	10/37 (27)	29/98 (30)
**Physical signs at admission**				
***Temperature*** †	*36.5 (36, 37.4)*	*38 (36.9, 38.4)*	*37.2 (36.5, 38.3)*	*37.5 (36.5, 38.2)*
**Temperature>38°C**	5/32 (16)	68/135 (50)	36/86 (42)	109/253 (43)
***Respiration rate*** †	*51 (40, 64)*	*30 (25, 40)*	*28 (24, 36)*	*32 (25, 40)*
**Elevated respiration rate** #	24/30 (80)	90/120 (75)	50/75 (67)	164/225 (73)
***Heart Rate*** †	*150 (130, 180)*	*112 (100, 120)*	*100 (82, 120)*	*110 (90, 128)*
**Elevated heart rate** §	31/37 (84)	113/148 (76)	47/92 (51)	191/277 (69)
***Systolic Blood Pressure*** †	*89 (79, 97)*	*110 (100, 130)*	*120 (100, 139.5)*	*110 (97, 130)*
**Low Systolic Blood Pressure** ¶	6/13 (46)	16/138 (12)	11/92 (12)	33/243 (14)
**Crackles**	19/30 (63)	121/132 (92)	70/80 (88)	210/242 (87)
**Wheezing**	17/27 (63)	39/88 (44)	40/64 (63)	96/179 (54)

* Unless otherwise stated.

† Median (IQR).

# Elevated respiration rate was defined as more than 40 breaths per minute for patients less than 5 years old and 25 breaths per minute for patients 5+ years old.

§ Elevated resting heart rate was defined as more than 120 beats per minute for patients less than 5 years old and 100 beats per minute for patients 5+ years old.

¶ Low systolic tension was defined as <90 mmHg.

**Table 3:** Selected hematology at time of admission by age group in influenza A H1N1pdm confirmed fatalities (excluding patients with influenza A H1N1pdm nosocomial infections unless otherwise reported), Argentina, June 15–July 31, 2009.

Hematology				
**Leukopenia** **(<4,500 cells/ml)**	8/31 (26)	38/124 (31)	20/85 (24)	66/240 (28)
**Leukocytosis** **(>11,000 cells/ml)**	13/31 (42)	25/124 (20)	30/85 (35)	68/240 (28)
**Lymphopenia** **	15/21 (71)	56/71 (79)	38/41 (93)	109/133 (82)
**Anemia** ††	28/41 (68)	66/162 (41)	44/99 (44)	138/302 (54)
**Thrombocytopenia** **(<150,000 cells/ml)**	6/25 (24)	40/81 (49)	19/53 (36)	65/159 (41)

Chemistry				
**Elevated Urea** **(>42 mg %)**	9/19 (47)	40/99 (40)	51/70 (73)	100/188 (53)
**Elevated AST (>80 UI/ml)**	7/13 (54)	26/55 (47)	16/35 (46)	49/103 (48)
**Elevated ALT (>80 UI/ml)**	5/13(38)	14/56 (25)	8/31 (26)	27/100 (27)

Acid-base				
**O_2_ Saturation <96%**	19/32 (59)	125/146 (86)	78/92 (85)	222/270 (82)
**pH<7.36**	15/20 (75)	41/102 (40)	40/77 (52)	96/199 (48)
**Elevated pCO_2_** **(>44 mmHg)**	10/19 (53)	18/101 (18)	22/77 (29)	50/197 (25)
**Acidosis or** **Hypoxemia** §§	23/33 (70)	132/147 (90)	86/94 (92)	241/274 (88)

** Lymphopenia was defined as <3000 cells/ml for patients under 5 years old, <2000 cells/ml for patients 5-12 years old and <1,500 cells/ml for patients over 12 years old.

†† Anemia was defined as less than 11 g/dl of hemoglobin (Hb) for patients less than 5 years old and for pregnant women (>15 years old), less than 11.5 g/dl Hb for patients 5–12 years old, less than 12 g/dl Hb for patients 12–15 years old and for non-pregnant women (>15 years old), and less than 13 g/dl Hb for men 15 years and older.

§§ “Acidosis or hypoxemia” is defined as pH under 7.36 or oxygen saturation less than 96%.

Among 176 patients with admission radiographs, (79%) had bilateral chest infiltrates, 71 (40%) had consolidation, 61 (35%) had interstitial pattern, 37 (21%) had both, and six (3%) had other findings (Table 4). For 236 (71%) of the 332 patients (including those with nosocomial infections), the primary hospital admission diagnosis was pneumonia. Pneumonia, however, only accounted for 54% of the primary diagnoses among children aged <5 years, with other respiratory illnesses accounting for over one third of the admission diagnosis among this age group.

**Table 4:** Selected X-ray patterns at time of admission by age group in influenza A H1N1pdm confirmed fatalities (excluding patients with influenza A H1N1pdm nosocomial infections unless otherwise reported), Argentina, June 15^th^–July 31^st^, 2009.

X-Rays patterns at admission				
**Consolidation only**	14/26 (54)	37/93 (40)	20/57 (35)	71/176 (40)
** Bilateral**	12/14 (86)	26/36 (72)	7/20 (35)	45/70 (65)
**Interstitial only**	8/26 (31)	29/93 (31)	24/57 (42)	61/176 (35)
** Bilateral**	8/8 (100)	28/29 (97)	23/24 (96)	59/61 (97)
**Consolidation + Interstitial**	3/26 (12)	23/93 (25)	11/57 (19)	37/176 (21)
** Bilateral**	3/3(100)	21/23 (91)	10/11 (91)	34/37 (92)

Primary Diagnosis at admission (includes patients that developed influenza A H1N1pdm nosocomial infections)				
**Pneumonia**	26/48 (54)	133/179 (74)	78/105 (74)	237/332 (71)
**Other respiratory diagnosis** ¶¶	16/48 (33)	28/179 (16)	21/105 (20)	65/332 (20)
**No record of any respiratory diagnosis** ***	6/48 (10)	18/179 (13)	6/105 (6)	30/332 (9)

¶¶ COPD, Asthma, atelectasis, tuberculosis, respiratory failure.

*** Fever syndrome, septic shock, leukemia, gastrointestinal problems, pregnancy, HIV/AIDS.

Of 304 patients admitted for influenza A H1N1pdm-associated illness (excluding nosocomial infections), the median time from symptom onset to admission was five days (IQR, 3–7 days). Most patients (299 [95%] of 315) needed ICU admission and, 292 were mechanically ventilated for a median of six days (IQR, 2–12 days). Among the 252 patients with available information on the start date of their mechanical ventilation, 207 (82%) were mechanically ventilated within the first 24 hours of admission to intensive care (Table 5).

**Table 5:** Treatment and Clinical course of 332 influenza A H1N1pdm-confirmed fatalities, June 15^th^–July 31^st^, 2009.

	<5 years	5–49 years	>50 years	Total
	N = 48	N = 179	N = 105	N = 332
Antiviral use (ATV)	N (%) total			
**Received ATV treatment**	40/48 (83)	156/179 (87)	94/105 (90)	290/332 (87)
**<48 hr after onset (early treatment)**	0/36 (0)	23/139 (17)	12/79 (15)	33/253 (13)
**<48 hr after first doctor visit**	6/30 (20)	56/112 (50)	43/68 (63)	105/210 (50)
**<48 hr after admission**	18/33 (55)	103/129 (80)	62/79 (78)	183/241 (76)
**Intensive care**				
**ICU admission**	46/47 (98)	162/170 (95)	91/98 (93)	299/315 (95)
**Mechanical ventilation (MV)**	47/47 (100)	160/174 (92)	85/99 (86)	292/320 (91)**
**Median No. Days: ICU to MV (IQR)**	0 (0–1)/38	0 (0–0)/140	0 (0–0)/73	0 (0–0)/233
**Median No. Days on MV (IQR)**	7 (4–13)/41	6 (2–12)/152	5 (2–11)/81	6 (2–12)/252
**Timelines Median Days (IQR)/N** ***				
** Onset—First doctor visit**	1 (0–3)/31	2 (0–4)/116	3 (1–5)/75	2 (0–4)/222
** First doctor visit—ATV treatment**	6 (3–10)/29	2 (0–5)/103	1 (0–5)/64	2 (0–5)/196
** Onset—Hospitalization**	6 (2–9)/27	4 (3–6)/125	5 (3–7)/79	5 (3–7)/231
** Hospitalization—ATV treatment**	1 (0–4)/27	0 (0–2)/118	0 (0–1)/73	0 (0–2)/218
** Hospitalization—ICU**	0 (0–2.5)/32	1 (0–2)/127	0 (0–1)/75	0 (0–1)/234
** Onset—Death**	15 (8.5–22)/32	12 (7–20.5)/140	13 (7–20)/87	13 (7–20)/259

** eleven case-patients were ventilated outside of the ICU because of space-limitations.

*** excluding influenza A H1N1pdm nosocomial infections.

The most frequent organ failure was respiratory failure (97%). Additionally, 85% of the patients met the criteria for acute respiratory distress syndrome (ARDS) during their hospitalization. These patients had hypoxemia [median Pa/FiO2 87 (IQR: 60–129)], hypercapnia [median PaCO2 54 (IQR: 43–72), and acidosis [median pH 7.24 (IQR: 7.10–7.33) requiring elevated concentration of oxygen (FiO2 100% in 72% of cases), maximal high positive end expiratory pressure [median 15 (IQR: 10–18)] and use of ventilation in prone position or alveolar recruitment maneuvers in 41% of patients. Hemodynamic failure requiring vasoactive drugs occurred in 54% of patients. Renal failure was present in 134 (43%) of the 312 patients with available data and 29% of these required dialysis. Of the 134 patients with renal failure, 31 (23%) had previous chronic renal failure of which eight [6%] were on chronic hemodialysis. Six (13%) of 47 children aged <5 years with available data developed renal failure compared to 128 (48%) of 265 persons aged ≥5 years (p<0.0001).Hematologic failure was diagnosed in 44%, hepatic failure in 5% and multiorgan failure in 71% of influenza A H1N1pdm fatalities.

Of all 332 patients, 290 (87%) received antiviral treatment; 183 (76%) ≤2 days of admission but only 33 (13%) of 253 with information on timing of administration received antivirals ≤48 hours of symptom onset. None of the 48 fatalities aged <5 years received antivirals ≤48 hours of symptom onset; 20% of these children received antivirals ≤48 hours of a doctor's visit, and only 55% received antivirals ≤48 hours of hospitalization compared to at least 78% in older age groups (Table 5).

**Figure 2:**
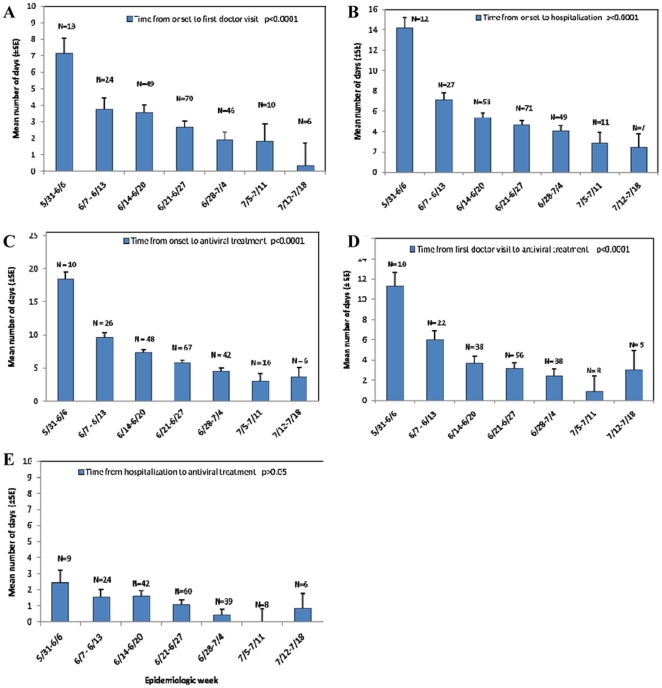
Mean number of days from date of onset to date of first doctor visit (A), hospitalization (B), and antiviral treatment (C); Mean number of days from first doctor visit (D) and hospitalization (E) to antiviral treatment among influenza A H1N1pdm fatalities by epidemiological week of onset.

As the pandemic progressed, the mean time from symptom onset to first doctor visit and symptom onset to hospitalization among fatalities decreased (p<0.001). Similarly, the mean time from symptom onset to antiviral treatment decreased from 19 days during epidemiologic week 22 to four days during week 28 (p<0.001). The time from the first doctor visit to antiviral treatment also decreased as the pandemic progressed (p<0.001) while the time from hospitalization to antiviral treatment remained brief (Figure 2A–E).

At least one comorbidity [11] was reported in 75% of cases with such information in their charts. The most common comorbidities were chronic pulmonary disease (including asthma) in 28%, metabolic disorder (including diabetes) in 25%, and immunosuppression in 24% of fatalities. Among children aged <5 years, chronic pulmonary disease (42%) was also the most common comorbidity followed by neonatal pathologies (i.e. genetic disorders, congenital malformations, and preterm births) (35%) and neurologic disease (19%) (Table 6). The prevalence of asthma was low among fatalities (6%). In contrast, 4% of patients were HIV positive, a proportion higher than the 0.4% country prevalence [12]. Similarly, 32% of fatalities were obese, a proportion higher than the 15% country prevalence [13].

**Table 6:** Comorbidities* and underlying conditions among influenza A H1N1pdm confirmed fatalities by age groups, Argentina, June 15^th^–July 31^st^, 2009.

Age groups	<5	5–49	50+	Total
	N = 48	N = 179	N = 105	N = 332
Comorbidities *	N (%) total			
**Chronic pulmonary disease** †	20/48 (42)	29/152 (19)	33/94 (35)	82/294 (28)
**Asthma**	0/41 (0)	11/142 (8)	5/89 (6)	16/272 (6)
**Cardiovascular disease** §	5/41 (12)	16/154 (10)	35/94 (37)	56/289 (19)
**Renal disease**	2/41 (5)	11/151 (7)	18/88 (21)	31/280 (11)
**Hepatic disease**	1/39 (3)	8/151 (5)	4/85 (5)	13/275 (5)
**Hematological disease**	3/38 (8)	22/148 (15)	8/88 (9)	33/274 (12)
**Metabolic disorder** ¶	3/42 (7)	34/156 (22)	35/91 (39)	72/289 (25)
**Diabetes**	0/40 (0)	22/150 (15)	22/87 (25)	44/277 (16)
**Immunosuppression** #	4/42 (10)	43/163 (26)	24/92 (26)	71/297 (24)
**HIV/AIDS**	0/38 (0)	10/155 (7)	1/85 (1)	11/278 (4)
**Other comorbidities** **	29/48 (60)	34/160 (21)	12/93 (13)	75/301 (25)
**Neurologic disease** ††	9/48 (19)	33/160 (21)	12/93 (13)	54/301 (18)
**Neonatal pathologies**	17/48 (35)	n/a	n/a	n/a
**At least 1 comorbidity** *	39/48 (81)	115/170 (68)	85/101 (84)	239/319 (75)

Underlying conditions				
**Obesity**	0/40 (0)	54/154 (35)	34/82 (42)	88/276 (32)
**Hypertension**	1/40 (3)	28/153 (18)	58/94 (62)	87/287 (30)
**Smoking** §§	n/a	35/171 (20)	29/103 (28)	64/321 (20)
**Alcohol dependence**	n/a	9/145 (6)	15/86 (17)	24/269 (9)
**Substance abuse**	n/a	6/142 (4)	2/81 (3)	8/261 (3)
**Pregnancy/postpartum** ¶¶	n/a	20/91 (22)	0/44 (0)	20/155 (13)

* Comorbidities as defined by the Advisory Committee on Immunization Practices ^10^.

† Including asthma.

§ Excluding hypertension.

¶ Including diabetes.

# Persons who have immunosuppression (including immunosuppression caused by medications or by human immunodeficiency virus, HIV).

** Persons who have any condition (e.g., cognitive dysfunction, spinal cord injuries, seizure disorders, or other neuromuscular disorders) that can compromise respiratory function or the handling of respiratory secretions or that can increase the risk for aspiration.

†† Including cerebrovascular disease, cerebral palsy, epilepsy, down syndrome, neurochronic disease.

§§ Does not include former and passive smokers.

¶¶ Women only.

**n/a** not applicable.

We identified 16 pregnant women and four postpartum women among fatalities. Four of the pregnant women were in their second trimester while 11 were in their third trimester. Eight delivered by caesarean section after being admitted to the ICU, two whom had stillbirths. Pregnant women had significantly longer ICU stays than non-pregnant women aged 15–44 years (11 vs. 4 days, p = 0.01). One pregnant and two postpartum women did not receive any antiviral treatment during the course of their illness. Among the 17 pregnant or postpartum women that received antivirals, none received them within the recommended 48 hours after illness onset but a median of seven days (IQR: 5–9 days) later. Fifty percent received antivirals <1 day after hospitalization (Table 7).

**Table 7:** Description of pregnant, postpartum and fertile age women among influenza A H1N1pdm confirmed deceased patients, Argentina, June 15^th^–July 31^st^, 2009.

	Pregnant andPostpartum (N = 20)	Non-pregnant(N = 44)
**Median age (range)**	26.5 (19–41)	32.5 (16–44)
**Postpartum at symptom onset**	4	n/a
**Pregnant at symptom onset**	16	n/a
* Second trimester*	4/15	n/a
* Third trimester*	11/15	n/a
**Stillbirths**	2/16	n/a
**Delivered after ICU admittance**	8/16	n/a
* C-sections*	13/14	n/a
**Comorbidities**		
* Chronic pulmonary disease*	1/15	9/40 (23%)
* Cardiovascular disease*	1/17	3/39 (8%)
* Renal disease*	0/16	2/40 (5%)
* Hepatic disease*	0/16	4/39 (10%)
* Hematological disease*	3/18	3/37 (8%)
* Metabolic disorder (diabetes)*	3/18	16/39 (41%)
* Immunosuppression*	1/18	12/41 (29%)
* Other condition*	2/18	4/39 (10%)
* At least 1 condition*	9/19	29/42 (70%)
* Obesity*	2/16	16/38 (42%)
* Hypertension*	2/16	7/40 (18%)
**Antiviral (ATV) treatment**	17/20	39/44 (89%)
** <48hrs from symptom onset**	0/17	4/29 (14%)
**Timelines: Median Days (IQR)**		
**Onset—ATV treatment**	7 (5–9)	5 (3–7)
**First doctor visit—ATV treatment**	4 (2.5–6)	1 (0–5)
**Hospitalization—ATV treatment**	1 (1–3)	0 (0–2)
**Onset —Hospitalization**	4 (2–6)	4 (2–5)
**ICU— Death**	11 (6–16)	4 (2–12)
**Onset— Death**	14 (13–17)	9 (5–18)

**n/a** not applicable.

## Discussion

Most fatal cases presented to the hospital with acute severe respiratory compromise or ARDS and required immediate mechanical ventilation with respiratory failure as their main cause of death. The median age of fatalities was consistent with those found in other influenza A H1N1pdm studies [14], [15], [16], [17], [18], [19]. Three-quarters of confirmed influenza A H1N1pdm fatalities occurred among persons aged <50 years, however, the highest incidence of H1N1pdm fatalities occurred among persons aged 50–65 years and children <5 years (half of them <1 year).

Two-thirds of all cases had at least one comorbidity as defined by ACIP [11]. In addition, healthy pregnant women were disproportionately affected as has been found in other studies [2], [20], [21], [22]. The unusually high proportion of decedents who were obese in our case-series (i.e. 33%) is consistent with studies [2] [23], [24] that suggest that obesity may be a risk factor for severe influenza disease. Although our study did not determine which fatalities had acquired immunodeficiency syndrome or were on antiretroviral therapy, we found a higher prevalence of influenza A H1N1pdm co-infection among HIV positive fatalities when compared to the general population. Such data are important because early reports suggested that HIV-infected individuals might experience more severe complications from influenza A H1N1pdm infection [20], [24], [25], [26], a finding not confirmed in later studies [27], [28], [29].

Even though the government of Argentina recommended oseltamivir treatment to high-risk persons [30], fewer than 15% of fatalities received antiviral treatment during the recommended 48 hours after symptom onset and only half within 48 hours of a physician visit. No children aged <5 years received early treatment, and only 20% received oseltamivir within 48 hours of the physician visit. Although a previous case-series of 251 children infected with influenza A H1N1pdm with 13 fatalities did not find a difference between prompt consultation, ICU admission, survival, and oseltamivir treatment [3], we recommend early treatment based on the findings of larger case-series [31]. The apparent reluctance to provide early antivirals to young children may have been caused by the clinician's lack of familiarity with the use of oseltamivir and their concerns over potential adverse events among very young children. Indeed, at the onset of the pandemic, clinicians were required to provide antiviral treatment to children aged <1 under strict medical supervision and under hospital supervision to children aged <3 months [30].

The high proportion of pregnant or postpartum women in our case-series of decedents is concordant with reports that identify pregnancy as a risk factor for severe influenza illness and death [21], [32], [32], [34], [35]. During the pandemic, pregnant women were identified as high-risk and the government of Argentina approved social distancing measures to protect them such as strongly urging them to stay home from work with paid leave. We observed that <50% of pregnant women had comorbidities, compared to 70% of influenza A H1N1pdm infected non-pregnant women and 80% of pregnant women in the U.S. [34]. In our study, these predominantly healthy pregnant and postpartum women received antivirals a median of seven days after symptom onset compared to five days among non-pregnant women. Such finding that suggests that although antiviral treatment ≤4 days after symptom onset was associated with milder influenza A H1N1pdm disease [34], clinicians may have been hesitant to use them possibly due to lack of familiarity with the use of oseltamivir and their concerns over potential adverse events among pregnant women and their unborn children.

Although other studies have shown a high frequency of dyspnea in critically ill patients, our analyses demonstrates our case-patients also frequently had tachypnea (73%), tachycardia (69%) and acidosis and/or hypoxemia (88%) on admission. Gastrointestinal symptoms were more frequently reported than in adults infected with seasonal influenza but not as frequently as in other influenza A H1N1pdm studies [14], [20], [21], [36]. Thrombocytopenia upon admission was frequently observed among decedents, and was more prevalent than previously reported among patients with influenza A H1N1pdm infection [37], [38], [39].

During the pandemic, an insufficient number of ICU beds were available for all patients. Critically ill patients occasionally received mechanical ventilation outside the ICU, at the emergency department or special facilities [40], [41]. In most patients, ARDS with respiratory failure was the leading cause of death. This is in contrast to ARDS caused by other etiologies where respiratory failure accounts for <20% of deaths [42], [43], [44]. This finding has also been observed in other influenza A H1N1pdm studies [2], [45], [46]. Acute renal failure requiring dialysis was observed frequently among patients aged ≥5.

Our analysis indicates that time from symptom onset to clinic visit and to hospitalization decreased as the pandemic progressed. Time to antiviral treatment also decreased during the first two weeks of the study. These data suggest that affected persons sought care earlier and physicians used antivirals more rapidly as the pandemic progressed and may explain why the case fatality proportion of hospitalized patients decreased from a peak at the beginning of the pandemic. The initial delay in health seeking may have been caused by high risk persons that did not suspect that they had influenza illness or that were not aware of the need to seek care within 48 hours of symptom onset when antivirals are most effective. The initial delay in treatment could have been caused by unavailability of antivirals or by primary care physicians unfamiliar with their use. In Argentina, only a small stockpile of oseltamivir was available at the beginning of the pandemic which was insufficient to treat all patients once influenza A H1N1pdm spread throughout the country.

Our study has several limitations. Our case-series design did not allow us to collect information from controls to further substantiate whether potential risk factors were indeed associated with death from H1N1pdm. The data obtained was limited to non-standardized medical records and there was no opportunity to confirm details with family members. Due to the limited availability of laboratory testing during the study period, the number of confirmed fatalities represented only about half of the currently confirmed fatalities and may not be representative of other fatalities during this period or of those who occurred at home. Moreover, we only assessed hospitalized laboratory-confirmed influenza A H1N1pdm fatalities for which we had medical records, and these cases might have manifested differently to those occurring at home or those not tested for influenza. Laboratory samples and tests did not follow a study protocol but represented the choices made by clinicians during the management of patients. Our study did not include autopsy data. Several parameters that define risk factors and underlying conditions, such as height and weight data, smoking history and alcohol consumption, were not available for all case-patients. We were unable to compare risk factors for death compared to severe disease and the effect of antiviral treatment as we did not review charts from surviving hospitalized patients.

After the study was conducted, Argentina has emphasized protecting young children and pregnant women. The 2010 monovalent influenza A H1N1pdm influenza vaccination campaign in Argentina aimed at a 95% coverage among pregnant women and 85% coverage among children aged <4 years [47]. Continued efforts should be made to promote influenza vaccination among groups at high risk of complications from influenza infection, ensure national surge capacity for intensive care and mechanical ventilation, secure antiviral stocks, guarantee their availability and accessibility, and to provide guidelines to health care providers on appropriate and timely use especially in vulnerable populations.

## References

[1] Dawood FS, Jain S, Finelli L, Shaw MW, Lindstrom S (2009). Emergence of a novel swine-origin influenza A (H1N1) virus in humans.. N Engl J Med.

[2] Estenssoro E, Rios FG, Apezteguia C, Reina R, Neira J (2010). Pandemic 2009 influenza A in Argentina: a study of 337 patients on mechanical ventilation.. Am J Respir Crit Care Med.

[3] Libster R, Bugna J, Coviello S, Hijano DR, Dunaiewsky M (2010). Pediatric hospitalizations associated with 2009 pandemic influenza A (H1N1) in Argentina.. N Engl J Med.

[4] Clarín (2009). Una beba de tres meses es la primera víctima fatal de Gripe A en la Argentina. .. http://edant.clarin.com/diario/2009/06/15/um/m-01939573.htm.

[5] Ministerio de Salud Presidencia de la Nación (2009).

[6] PAHO (2006). PAHO-CDC Generic Protocol for Influenza Surveillance.. http://www.paho.org/English/AD/DPC/CD/flu-snl-gpis.pdf.

[7] CDC (2009). Protocol of realtime RTPCR for Influenza A (H1N1).. http://www.who.int/csr/resources/publications/swineflu/CDCRealtimeRTPCR_SwineH1Assay-2009_20090430.pdf.

[8] Levy MM, Fink MP, Marshall JC, Abraham E, Angus D (2003). 2001 SCCM/ESICM/ACCP/ATS/SIS International Sepsis Definitions Conference.. Crit Care Med.

[9] Bernard GR, Artigas A, Brigham KL, Carlet J, Falke K (1994). The American-European Consensus Conference on ARDS. Definitions, mechanisms, relevant outcomes, and clinical trial coordination.. Am J Respir Crit Care Med.

[10] PAHO (2009). Regional Update: Pandemic (H1N1).. http://new.paho.org/hq/dmdocuments/2010/Regional_update%20EW%2014.pdf.

[11] Fiore AE, Shay DK, Broder K, Iskander JK, Uyeki TM (2009). Prevention and control of seasonal influenza with vaccines: recommendations of the Advisory Committee on Immunization Practices (ACIP).. MMWR.

[12] Ministerio de Salud Presidencia de la Nación (2009). Boletín sobre el VIH-SIDA en la Argentina. 26.. http://www.msal.gov.ar/sida/pdf/boletines-inves-publi/boletin-12-09.pdf.

[13] Ministerio de Salud Presidencia de la Nación (2006). Primera Encuesta Nacional de Factores de Riesgo. Informe de Resultados. Peso Corporal.. http://msal.gov.ar/ent/VIG/Publicaciones/Encuestas_Poblacionales/PDF/Encuesta%20NNacional%20De%20Factores%20De%20Riesgo%202005%20-%20Version%20Breve.pdf.

[14] Ministerio de Salud de Chile (2010). Plan Nacional de Preparación y Respuesta para una Pandemia de Influenza: Actualización 2010.. http://epi.minsal.cl/epi/html/secciones/Pandemia2009/PlanPandemiaMINSAL2010.pdf.

[15] Ministerio de Salud Pública y Bienestar Social (2010). Boletín semanal de la situación epidemiológica, Paraguay.. http://www.vigisalud.gov.py/attachments/069_2010-02-05%20SE%2005%20(Parte%20Influenza%20Pand%C3%A9mica%20Nro%2085).pdf.

[16] Ministerio de Salud Pública (2009). Informe Final Influenza A (H1N1)v, Uruguay - Diciembre 2009.. http://www.msp.gub.uy/ucepidemiologia_4027_1.html.

[17] CDC (2009). Use of influenza A (H1N1) 2009 monovalent vaccine: recommendations of the Advisory Committee on Immunization Practices (ACIP).. MMWR.

[18] Ministério da Saúde do Brasil (2010). Influenza Pandêmica (H1N1) 2009 – Análise da situação epi demiológica e da resposta no ano de 2009..

[19] Secretaria de Salud M (2010). Situación actual de la pandemia. 25-Enero-2010.. http://portal.salud.gob.mx/descargas/pdf/influenza/situacion_actual_epidemia_250110.pdf.

[20] Louie JK, Acosta M, Winter K, Jean C, Gavali S (2009). Factors associated with death or hospitalization due to pandemic 2009 influenza A(H1N1) infection in California.. JAMA.

[21] Jain S, Kamimoto L, Bramley AM, Schmitz AM, Benoit SR (2009). Hospitalized patients with 2009 H1N1 influenza in the United States, April-June 2009.. N Engl J Med.

[22] Webb SA, Pettila V, Seppelt I, Bellomo R, Bailey M (2009). Critical care services and 2009 H1N1 influenza in Australia and New Zealand.. N Engl J Med.

[23] Morgan OW, Bramley A, Fowlkes A, Freedman DS, Taylor TH (2010). Morbid obesity as a risk factor for hospitalization and death due to 2009 pandemic influenza A(H1N1) disease.. PLoS One.

[24] Kumar A, Zarychanski R, Pinto R, Cook DJ, Marshall J Critically ill patients with 2009 influenza A(H1N1) infection in Canada. Jama. 2009;302(17):1872-9..

[25] CDC (2009). Updated Interim Recommendations—HIV-Infected Adults and Adolescents: Considerations for Clinicians Regarding 2009 H1N1 Influenza.. http://www.cdc.gov/h1n1flu/guidance_hiv.htm.

[26] Archer B, Cohen C, Naidoo D, Thomas J, Makunga C (2009). Interim report on pandemic H1N1 influenza virus infections in South Africa, April to October 2009: epidemiology and factors associated with fatal cases.. Euro Surveill.

[27] Feiterna-Sperling C, Edelmann A, Nickel R, Magdorf K, Bergmann F (2010). Pandemic influenza A (H1N1) outbreak among 15 school-aged HIV-1-infected children.. Clin Infect Dis.

[28] Riera M, Payeras A, Marcos MA, Viasus D, Farinas MC (2010). Clinical presentation and prognosis of the 2009 H1N1 influenza A infection in HIV-1-infected patients: a Spanish multicenter study.. AIDS.

[29] Giannattasio A, Lo Vecchio A, Russo MT, Pirozzi MR, Barbarino A (2010). Pandemic flu: a comparative evaluation of clinical, laboratory, and radiographic findings in HIV-positive and negative children.. AIDS.

[30] ANMAT (2009). Uso de Oseltamivir y Zanamivir en menores de 1 ano y en embarazadas.. http://www.anmat.gov.ar/PUBLICACIONES/MEDICAMENTOS/RECOMENDACIONES_OSELTAMIVIR_ZANAMIVIR.PDF.

[31] Rodriguez-Noriega, Gonzalez-Díaz E, Morfín-Otero R, Briseño-Ramírez J, Perez-Gómez HR E (2010). Hospital triage system using an influenza-like illness scoring system during the (H1N1) pandemic 2009- Mexico. Jalisco, México Hospital Civil de Guadalajara, Fray Antonio Alcalde.. PLoS One.

[32] Louie JK, Acosta M, Jamieson DJ, Honein MA (2009). Severe 2009 H1N1 influenza in pregnant and postpartum women in California.. N Engl J Med.

[33] Siston AM, Rasmussen SA, Honein MA, Fry AM, Seib K (2010). Pandemic 2009 influenza A(H1N1) virus illness among pregnant women in the United States.. JAMA.

[34] Jamieson DJ, Honein MA, Rasmussen SA, Williams JL, Swerdlow DL (2009). H1N1 2009 influenza virus infection during pregnancy in the USA.. Lancet.

[35] ANZIC (2010). Critical illness due to 2009 A/H1N1 influenza in pregnant and postpartum women: population based cohort study.. BMJ.

[36] Lee EH, Wu C, Lee EU, Stoute A, Hanson H (2010). Fatalities associated with the 2009 H1N1 influenza A virus in New York city.. Clin Infect Dis.

[37] Dubnov-Raz G, Somech R, Warschawski Y, Eisenberg G, Bujanover Y (2011). Clinical Characteristics of Children with 2009 Pandemic H1N1 Influenza Virus Infections.. Pediatr Int.

[38] Ugarte S, Arancibia F, Soto R (2010). Influenza A pandemics: clinical and organizational aspects: the experience in Chile.. Crit Care Med.

[39] Venkata C, Sampathkumar P, Afessa B (2010). Hospitalized patients with 2009 H1N1 influenza infection: the Mayo Clinic experience.. Mayo Clin Proc.

[40] Raffo L (2009). [Influenza A(H1N1) epidemic in Argentina. Experience in a National General Hospital (Hospital Nacional Alejandro Posadas)].. Medicina (B Aires).

[41] Meites E, Farias D, Raffo L, Albalak R, Carlino OL (2011). Hospital Capacity during an Influenza Pandemic-Buenos Aires, Argentina, 2009.. Infect Control Hosp Epidemiol.

[42] Montgomery AB, Stager MA, Carrico CJ, Hudson LD (1985). Causes of mortality in patients with the adult respiratory distress syndrome.. Am Rev Respir Dis.

[43] Ferring M, Vincent JL (1997). Is outcome from ARDS related to the severity of respiratory failure?. Eur Respir J.

[44] Estenssoro E, Dubin A, Laffaire E, Canales H, Saenz G (2002). Incidence, clinical course, and outcome in 217 patients with acute respiratory distress syndrome.. Crit Care Med.

[45] Farias JA, Fernandez A, Monteverde E, Vidal N, Arias P (2010). Critically ill infants and children with influenza A (H1N1) in pediatric intensive care units in Argentina.. Intensive Care Med.

[46] Nin N, Soto L, Hurtado J, Lorente JA, Buroni M (2011). Clinical characteristics and outcomes of patients with 2009 influenza A(H1N1) virus infection with respiratory failure requiring mechanical ventilation.. J Crit Care.

[47] Ministerio de Salud Presidencia de la Nación (2010). Campaña Nacional de Vacunación para el Nuevo Virus de Influenza A H1N1 en Argentina: Manual del Vacunador Año 2010.. http://msp.rec.uba.ar/docs/ah1n1_2.pdf.

